# Fully Automatic Classification of Brain Atrophy on NCCT Images in Cerebral Small Vessel Disease: A Pilot Study Using Deep Learning Models

**DOI:** 10.3389/fneur.2022.846348

**Published:** 2022-03-24

**Authors:** Jincheng Wang, Sijie Chen, Hui Liang, Yilei Zhao, Ziqi Xu, Wenbo Xiao, Tingting Zhang, Renjie Ji, Tao Chen, Bing Xiong, Feng Chen, Jun Yang, Haiyan Lou

**Affiliations:** ^1^Department of Radiology, First Affiliated Hospital, School of Medicine, Zhejiang University, Hangzhou, China; ^2^State Key Laboratory of Medical Neurobiology and Collaborative Innovation Center for Brain Science, Institutes of Brain Science, Fudan University, Shanghai, China; ^3^Department of Neurology, First Affiliated Hospital, School of Medicine, Zhejiang University, Hangzhou, China; ^4^Taimei Medical Technology, Shanghai, China

**Keywords:** brain atrophy, linear measurement, automated classification, convolutional neural networks, cerebral small vessel disease, computed tomography, deep learning

## Abstract

**Objective:**

Brain atrophy is an important imaging characteristic of cerebral small vascular disease (CSVD). Our study explores the linear measurement application on CT images of CSVD patients and develops a fully automatic brain atrophy classification model. The second aim was to compare it with the end-to-end Convolutional Neural Networks (CNNs) model.

**Methods:**

A total of 385 subjects such as 107 no-atrophy brain, 185 mild atrophy, and 93 severe atrophy were collected and randomly separated into training set (*n* = 308) and test set (*n* = 77). Key slices for linear measurement were manually identified and used to annotate nine linear measurements and a binary classification of cerebral sulci widening. A linear-measurement-based pipeline (2D model) was constructed for two-types (existence/non-existence brain atrophy) or three-types classification (no/mild atrophy/severe atrophy). For comparison, an end-to-end CNN model (3D-deep learning model) for brain atrophy classification was also developed. Furthermore, age and gender were integrated to the 2D and 3D models. The sensitivity, specificity, accuracy, average F1 score, receiver operating characteristics (ROC) curves for two-type classification and weighed kappa for three-type classification of the two models were compared.

**Results:**

Automated measurement of linear measurements and cerebral sulci widening achieved moderate to almost perfect agreement with manual annotation. In two-type atrophy classification, area under the curves (AUCs) of the 2D model and 3D model were 0.953 and 0.941 with no significant difference (*p* = 0.250). The Weighted kappa of the 2D model and 3D model were 0.727 and 0.607 according to standard classification they displayed, mild atrophy and severe atrophy, respectively. Applying patient age and gender information improved classification performances of both 2D and 3D models in two-type and three-type classification of brain atrophy.

**Conclusion:**

We provide a model composed of different modules that can classify CSVD-related brain atrophy on CT images automatically, using linear measurement. It has similar performance and better interpretability than the end-to-end CNNs model and may prove advantageous in the clinical setting.

## Introduction

Cerebral small vessel disease (CSVD), a disorder of cerebral microvessels, is an expanding epidemic all over the world. CSVD causes approximately a quarter of ischemic strokes and most hemorrhagic strokes. It is the most common cause of vascular dementia, and worsens the resulting cognitive impairment, thus contributing to about 50% of dementias worldwide, a massive health burden in reality (1, 2). In the context of CSVD, brain atrophy is one of the important radiological descriptions, according to the standards for reporting vascular changes on neuroimaging (STRIVE) (3). The neuropathological basis includes neuron loss, cortical thinning, subcortical vascular disease with white matter thinning and contraction, arteriolar sclerosis, venous collagen degeneration, and secondary neurodegenerative changes (4). Brain atrophy usually appears with other signs of CSVD, and it is an important measure in imaging studies that are done to assess the burden of vascular damage in the brain (5–7). The path analyses show that the impact of CSVD-related lesions on the clinical status is partly due to brain atrophy and changes in cortical morphology (8). Atrophy is thought to mediate, at least partially, the effects of vascular lesions on cognition, and is mainly observed in patients with accelerating cognitive decline (9, 10). Brain atrophy is suggested to impair the potential for functional recovery after stroke. Severe atrophy thus has lower brain reserve, making it less resilient to ischemic injury (11, 12).

Non-contrast computed tomography (NCCT) has the advantages of broader acceptability, lower cost, and faster scanning speed (less motion artifacts) compared to magnetic resonance imaging (MRI), and it plays a fundamental role in neuroimaging (13). Brain atrophy, such as cortical atrophy and central atrophy, has characteristic findings on NCCT images. The typical sign of central atrophy is the enlargement of the ventricular system. Correspondingly, enlarged Sylvian fissures, widened sulci, and narrowed gyrus are markers of cortical atrophy (14). The visual assessment of these discoveries of brain atrophy is simple and convenient (15), but its accuracy depends on the higher professional knowledge and rich clinical experience of raters. Although some scales based on visual findings have been developed, it is relatively coarse, subjective, and might be prone to floor and ceiling effects. In view of the low tissue contrast on NCCT images, accurate tissue segmentation and volume calculation are still imperfect and are constantly being improved (16). The challenge is believed to be tougher for some CSVD patients (17). The presence of white matter hypoattenuation or white matter hypodensities on NCCT further reduces tissue contrast. One-dimensional linear measurement, a mature and reliable way to assess the brain atrophy, has been widely used in clinical practice (18–20). Previous studies have shown that one-dimensional linear measurement and three-dimensional measurement results have achieved substantial correlation (21, 22). It does not have high requirements for tissue contrast and can be used on either MRI or NCCT.

A significant progress has been made in the development of fully automated structural image analysis and computer-aided diagnosis technology (23). The fully automatic method provides several advantages, including the ability to complete the set tasks independently without manual operation, and the measurement results can be quantified and further analyzed. This method has been beneficially applied to patients with CSVD (24). As a branch of machine learning, deep learning can extract hidden features within big data automatically and has achieved advanced performance across various fields (25). On the one hand, the underlying mechanism of deep learning is still a black box (26), and some researchers are focused on visualization and are dedicated to improving the interpretability of the algorithms (27). Building the model pipeline following the human medical approach rather than generating an end-to-end model is also a potentially advantageous protocol in relation to certain tasks. Yet, for the brain atrophy of CSVD patients, automatic analysis is mostly performed on MRI. The establishment of a fully automatic evaluation model on NCCT images is a useful supplement with clinical value.

In this study, we aimed to develop a fully automated brain atrophy classification model based on NCCT images for CSVD subjects, using one-dimensional line measurements to overcome the limitations of low tissue contrast. Several clinical information such as age and gender, were also included in the model to improve the accuracy of the model (2D-integrated model). For performance comparison, an end-to-end model (3D model and 3D-integrated model) has also been developed based on a pre-trained 3D CNN algorithm.

## Materials and Methods

### Study Populations

**Figure 1:**
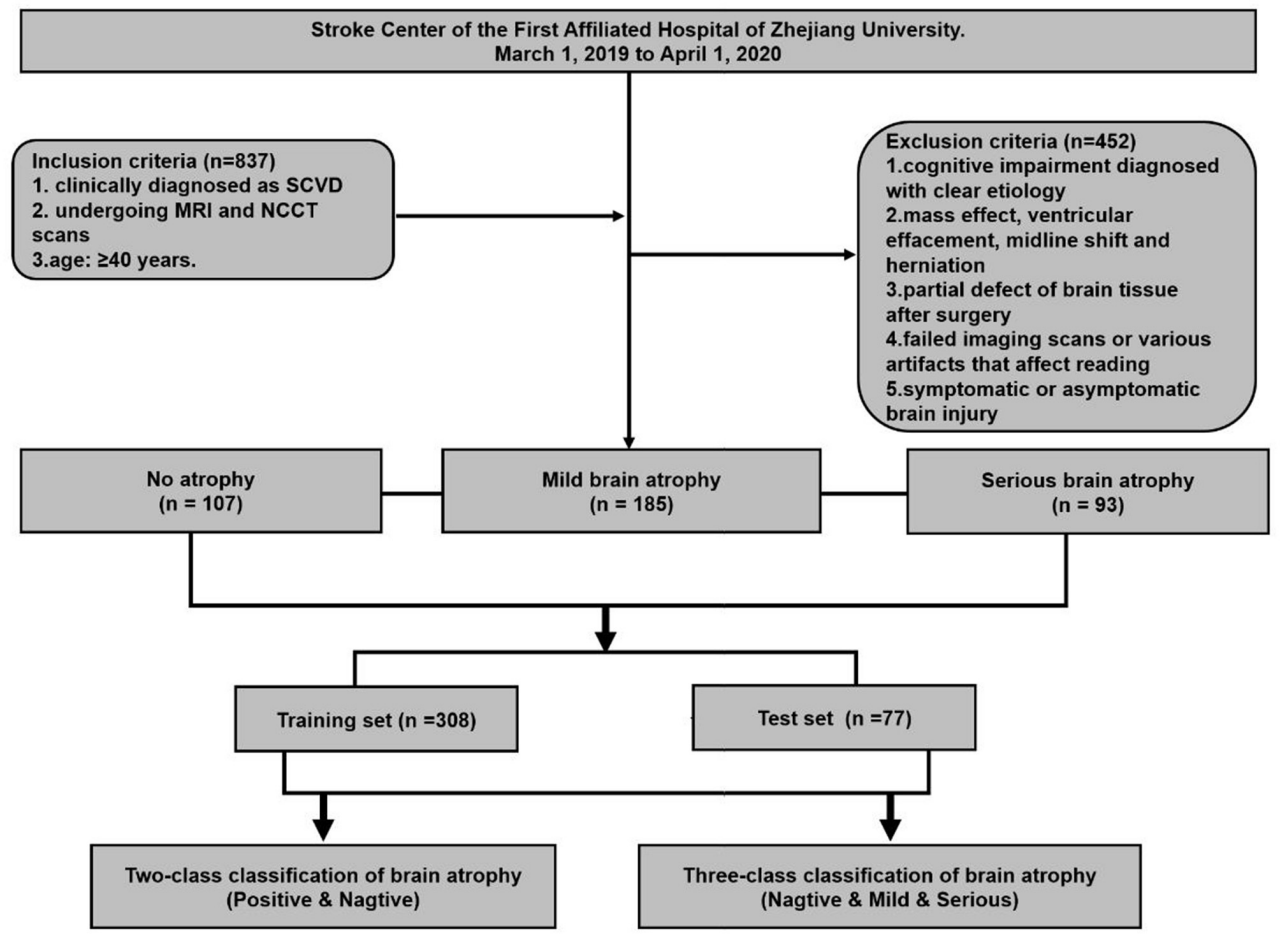
Flowchart of patient enrollment and strategy of training and testing for brain atrophy classification.

An observational study based on a cohort of outpatients and inpatients was conducted at the Stroke Center of the First Affiliated Hospital of Zhejiang University, from March 1, 2019 to April 1, 2020. This retrospective study was approved by the ethics committee of the first affiliated hospital of Zhejiang University (approval number: 2019.1511). A total of 385 eligible patients were enrolled in this study. The inclusion criteria were set as follows: (1) Patients who have been clinically diagnosed as CSVD, and at least one of the following neuroimaging markers on MRI images: white matter hyperintensities, recent small subcortical infarcts, lacunes, enlarged perivascular spaces, cortical microinfarcts, cerebral microbleeds, and brain atrophy (8). (2) The both MRI and NCCT head/brain scans of patients were used to make a set of MRI–NCCT pairs for accurate training preparations, and the time interval between the MRI and NCCT scans were required to be <3 months to avoid differences in brain structures due to possible disease progression. (3) Age equal to or >40 years. The following patients were excluded: (1) cognitive impairment diagnosed with clear etiology, such as poisoning, infection, degeneration diseases (Parkinson's disease, multiple system atrophy, corticobasal degeneration, and dementia with Lewy bodies, etc.), and immune demyelination (multiple sclerosis, Balo's concentric sclerosis, etc.); (2) patients with any indications that may affect the linear measurement, such as of intracranial mass effect and local mass effect, ventricular effacement, midline shift, and herniation; (3) partial defect of brain tissue after surgery; (4) failed imaging scans or various artifacts that affect reading; and (5) symptomatic or asymptomatic brain injury. The study flowchart is shown in Figure 1.

### Computed Tomography Image Dataset

All CT scans were performed using 64-rows helical CT scanners(Brilliance 64, Philips; LightSpeed VCT, GE Medical System) with a tube voltage of 20 kVp and a tube current of 180–200 mAs. The raw data of the Minimum slice thickness in the *Z* axis direction were 0.625 mm, and the reconstruction slice thickness was 5 mm with an image matrix of 512 × 512 pixels.

### Data Pre-Processing

Each image was normalized to the same intensity range 0–255 (WW = 80 HU, WL = 40 HU) and when deemed necessary the brain midline was rotated vertically. For 2D model training, all images were resampled to 224 × 224 pixels to reduce the GPU memory usage.

### Model Training and Testing

All NCCT scans were randomly separated into a training set (*n* = 308) and a test set (*n* = 77) for both two-type and three-type classification tasks. At first, all images were corrected by rotation on the X–Y plane, asserting the brains were perpendicular to the horizontal edges of image in each sample. A series of common data augmentation approaches were performed to increase training data diversity and size, such as random rotation (from −5 to 5 degrees), random shift, random zoom, left–right flipping, and adding Gaussian noise. We applied five-fold cross validation in the training set (the training set was randomly split into five subsets. A model was trained using the first four subsets, and then validated on the remaining subset. Five iterations were performed using each subset as validation set to optimize model parameters and to avoid overfitting. All models were trained only on the training set, and the testing set was reserved for performance evaluation of our models.

### Ground Truth Label (Reference for Model Training)

A neuroscientist and a radiologist, both with more than 20 years of personal experience, will reach a consensus on MRI–NCCT image pairs. The CSVD-related brain atrophy is labeled by the visual assessment on MRI (28). No enlargement of the ventricular system (third and fourth ventricles) and no enlargement of the sulci and lateral fissure is defined as no brain atrophy (*n* = 107). The significant enlargement of the ventricular system, along with significantly widened parietal lobe sulci (at least 2 sulci were >5 mm in width), and a significantly widened lateral fissure cistern is defined as severe atrophy (*n* = 93). The rest did not meet the above conditions and were regarded as having mild atrophy (*n* = 185). The high atrophy ratio was retained in the dataset to ensure sufficient positive samples for the effective learning process and evaluation of 3-classification algorithms. The demographic characteristics of the dataset are shown in Table 1.

**Table 1:** Demographic information of the study subjects.

	**No atrophy** **(***n*** = 107)**	**Mild atrophy (***n*** = 185)**	**Severe atrophy** **(***n*** = 93)**
Age (year)	48 (40 67)	74 (44 94)	81 (60 99)
Gender (male: female)	48:59	96:89	51:72

*Age presented as median (range)*.

### Manual Annotation on NCCT

**Figure 2:**
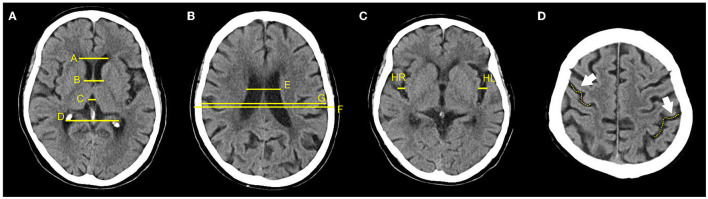
Annotation of 9 linear measurements and cerebral sulci enlargement. **(A–D)** were four key slices defied for linear measurements and Cerebral sulci enlargement identification. **(A)**, A, B, C, and D were manually annotated on basal ganglion slice showing most prominently the heads of the caudate nuclei (Key slice 1); **(B)**, E, F, and G were annotated on slice displaying most of the body of the lateral ventricle (key slice 2); **(C)**. HL and HR were manually annotated on slice a that the Sylvian fissures were most clearly visualized (key slice 3); **(D)**, Binary classification of cerebral sulci widening on the third slice above the top of lateral ventricle was also annotated (key slice 4).

The one-dimensional linear measurement was independently completed by another neuroradiologist on the four key slices of the NCCT images with ITK-SNAP software (US National Institutes of Health). This researcher was unaware of the label and clinical information of each subject. Results of the annotation were reviewed by the aforementioned two experts and a consensus was reached (Figure 2). Nine linear measurements included maximal frontal horn width (A), minimal intercaudate distance (B), maximal width of third ventricle (C), the choroid plexuses distance (D) on basal ganglion slice showing most prominently the heads of the caudate nuclei (key slice 1), minimal ventricular body width (E), maximal transversal intracranial width (F), and maximal transversal extracranial width (G) on a slice displaying most of the body of the lateral ventricle (key slice 2). The maximal width of Sylvian fissures (HR/HL) on the slice where measurement was most clearly visualized (key slice 3) (20, 29–31). It needs to be emphasized that enlargement of the parietal lobe sulci (at least 2 sulci being >5 mm in width) is a binary classification, implemented on the third slice above the top of the lateral ventricle (key slice 4), and still based on expert consensus as the ground truth.

### Overview of Pipeline Modules of Brain Atrophy Classification Model

**Figure 3:**
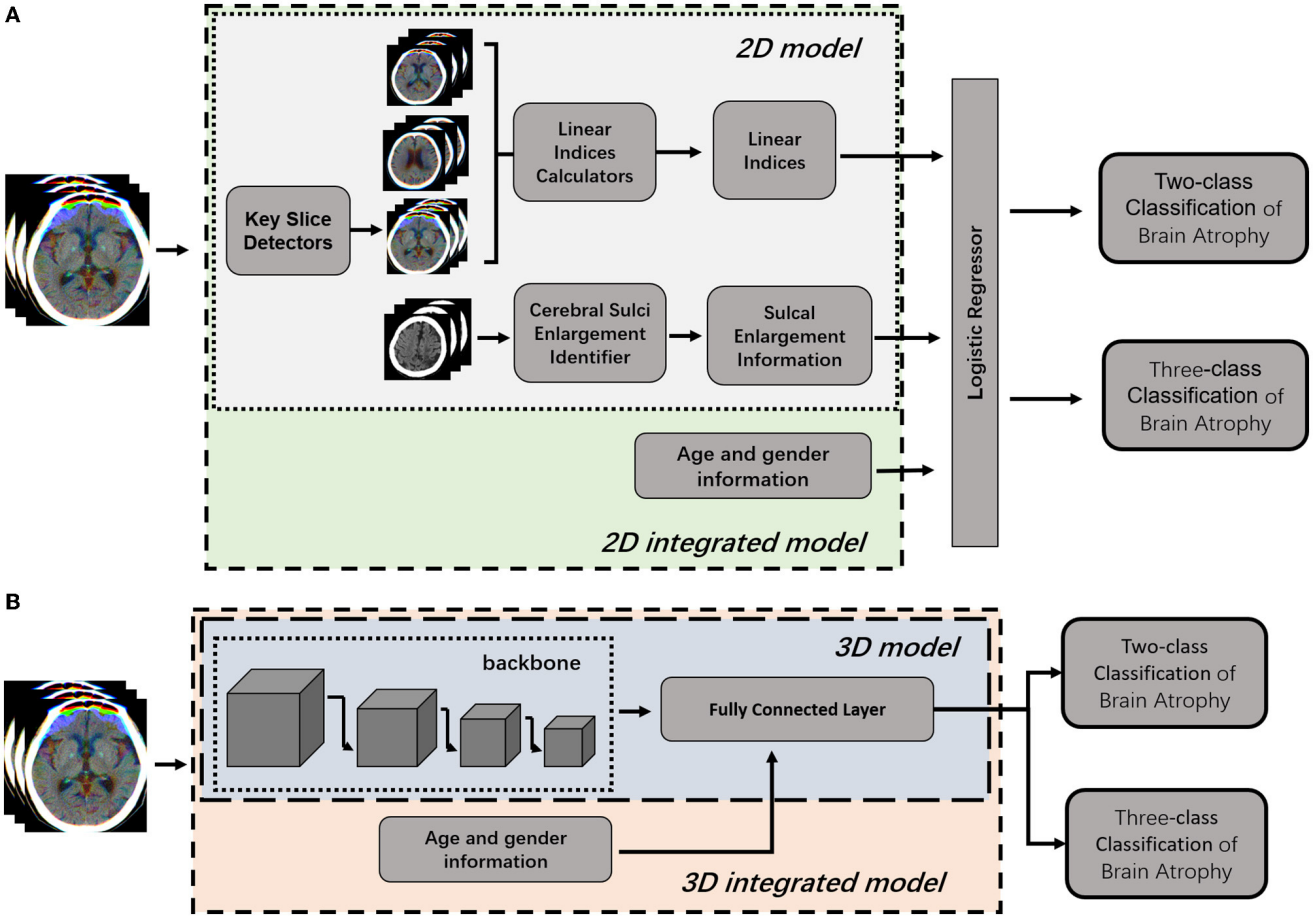
Illustration of 2D model and 3D model for brain atrophy classification. **(A)** 2D model was consisted of four modules, such as key slice detectors, linear measurements calculators, cerebral sulci enlargement identifier, and atrophy classifier. Information of age and gender were integrated into the atrophy classifier of 2D model to generate 2D-integrated model. **(B)** 3D model was developed based on a 3D CNN model. Information of age and gender were integrated into the full connected layer to generate the 3D-integrated model.

The pipeline of the classification model consists of four modules: (1) key slice detection module, (2) automatic measurement module, (3) identification module of cerebral sulci widening, and (4) classifier module (Figure 3A). Key slice detectors, linear measurement calculators and cerebral sulci enlargement identifiers were developed based on CNN algorithms, while the atrophy classifier was implemented by a logistic regression model. All included CT images after pre-processing were input into the pipeline: key slices were identified first, and then linear measurements and cerebral sulci information were automatically identified. Seven linear measurements such as the Huckman Number (A + B), ventricle index (D/A), lateral ventricular body index (F/E), width of lateral ventricular body index (G/E), ventricle forefoot index (G/A), maximum width of third ventricle (C), average width of Sylvian fissures ([HL + HR]/2) (20, 30, 31), along with cerebral sulci enlargement information were used to identify whether the subject had cerebral atrophy or not in the two-class classification, and no atrophy, mild atrophy, or severe atrophy in the three-class classification.

### Module 1: Key Slice Detection

The ResNet34, consisting of four convolutional-pooling blocks and a fully connected layer, was used as the backbone model to detect four defined key slices (32). To train the proposed CNN model, momentum was applied as the optimizer to minimize the cross entropy between annotated key slices and the prediction outputs. The batch size was set to eight and the learning rate was applied with piecewise-scheduled decay with warming starting. We selected models for each key slice with the best accuracy in the validation set to construct the detector for each slice.

### Module 2: Automatic Measurement

Models for nine linear measurements in key slices 1, 2, and 3 were developed based on the ResNet 18 backbone. As the structure and morphology of the brain are very similar on adjacent slices to the key slice, we fed the key slice and two adjacent slices into three channels of the RGB color image and generated a three-channel fused image to train the proposed model. We used the mean square error (MSE) as the cost function and Adam as the optimization algorithm. The batch size is 16, learning rate is 0.0001, and the value of decay is 0.8 with a decay step of 10,000.

### Module 3: Cerebral Sulci of Parietal Lobe Enlargement Identification

Model for cerebral sulci of parietal lobe enlargement identification in key slice 4 was developed based on ResNet 18 backbone. Three channel fused image consisting of key slice 4 and adjacent slices was used to train the classification model. We used cross entropy as the loss function and momentum as the optimization algorithm. The batch size was 8 and the learning rate was applied with piecewise-scheduled decay with warming starting.

### Module 4: Brain Atrophy Classification

Seven linear measurements and cerebral sulci enlargement information were used to train a logistic regression classifier for no atrophy/atrophy two-class classification or no atrophy/mild atrophy/severe atrophy three-class classification. Furthermore, linear measurements, cerebral sulci of parietal lobe enlargement information as well as age and gender were utilized to build logistic regression as an integrated model.

These four modules had different outputs which were inter-connected sequentially, and outputs from the last module were used as training inputs to the next module. The outputs of key slice detection module were the machine-selected four slices (key slice 1–4) from each subject's NCCT image set. The outputs of the automatic measurement module were eight straight lines automatically drawn by the machine and their length values (A-G, HR/HL). For better visualization, these lines were displayed on key slices in different colors. The sulcus enlargement recognition module outputted an automatic judgment result, with or without sulcus enlargement. The outputs of classifier module were obtained by the machine after integrating all the information; it could be binary classification (atrophy or no atrophy) or three classifications (no atrophy, mild atrophy, or severe atrophy).

### Overview of End-To-End 3D CNN Model

To compare with the 2D model, an end-to-end deep learning algorithm for two-class and three-class classification were trained using Med3D, a heterogeneous three-dimensional network pretrained on the 3DSeg-8 dataset was employed as the backbone (Figure 3B). The batch size was set to four and the Adam algorithm was utilized to optimize model parameters with a learning rate of 0.0001 in the backbone and 0.001 in fully connected layers. Optimization of model parameters was considered to converge once the model's performance on the validation set stopped improving for 50 epochs. Age and gender information were integrated into the fully connected layer to generate the 3D-integrated model. The outputs of 3D model or 3D-integrated model directly generated classification results, either a binary classification (atrophy or no atrophy) or three classifications (no atrophy, mild atrophy, or severe atrophy).

### Statistical Analysis

Paired *t*-test and Pearson correlation test were used to evaluate the performance of automatic linear measurements. The consistency of the cerebral sulci dichotomy was assessed on accuracy and Cohen's kappa. The model performance of two-class classification was evaluated by sensitivity, specificity, accuracy, F1 score, and area under the curve (AUC) with 95% *CI*. The 0.5 was set as the probability threshold of binarized class labels. The differences between Receiver Operating characteristic (ROC) curves were further analyzed using the DeLong test. For three-type classification, the model performance was evaluated based on accuracy, average F1 score, and weighted kappa. All statistical analyses were performed using the R programming language (version 3.6); the *p* < 0.05 was considered as statistically significant.

## Results

### Module Performance

The performance of three modules, key slice detection, linear measurements, and cerebral sulci enlargement identification, were evaluated in the test set. The detection accuracy for key slices 1, 2, 3, and 4 were 0.887, 0.558, 0.934, and 0.574, respectively, and they were compared with the annotated ground truth. The accuracy of the detected key slice and its two adjacent slices reached 1 for all key slices (Supplementary Table S1). The correlation results of nine linear measurements between annotated ground truth and automated measurements were listed in Table 2. In the test set, no significant difference was found in the mean value of each linear measurement between automated and manual annotations. Automated measurements achieved a very good correlation with ground truth in the minimal intercaudate distance, maximal transversal intracranial width, and maximal transversal extracranial width (correlation coefficient ≥ 0.8), a strong correlation in maximal width of the third ventricle, minimal ventricular body width, and maximal width of Sylvian fissures in left and right (correlation coefficient ≥ 0.6), and a moderate correlation in maximal frontal horn width and the choroid plexuses distance (correlation coefficient ≥ 0.4). For the cerebral sulci dichotomy, the accuracy of the deep learning algorithm was 0.818, and the Cohen's kappa was 0.636.

**Table 2:** Correlation analysis between automated measurement and manual ground truth for linear measurements.

	**GT**	**AI**	**t**	***p*** **-Value**	**PCC**	***p*** **-Value**
A	35.999 ± 4.186	35.936 ± 2.686	0.363	0.717	0.584	<0.001
B	19.001 ± 4.151	18.808 ± 3.172	1.524	0.128	0.803	<0.001
C	8.215 ± 2.350	8.014 ± 1.536	1.867	0.063	0.618	<0.001
D	52.977 ± 5.858	52.970 ± 3.743	0.026	0.979	0.537	<0.001
E	34.223 ± 6.559	34.147 ± 4.452	−0.16	0.873	0.779	<0.001
F	142.290 ± 6.484	142.579 ± 5.642	−1.854	0.064	0.881	<0.001
G	123.591 ± 7.593	123.862 ± 6.810	−1.894	0.059	0.93	<0.001
HL	6.083 ± 3.078	6.365 ± 3.233	0.523	0.602	0.68	<0.001
HR	6.365 ± 3.229	6.159 ± 2.489	1.729	0.085	0.695	<0.001

*GT, manual ground truth; AI, linear measurements achieved by 2D model; PCC, Pearson correlation coefficient*.

### Performance of the Two-Type Classification for Brain Atrophy With CSVD

**Figure 4:**
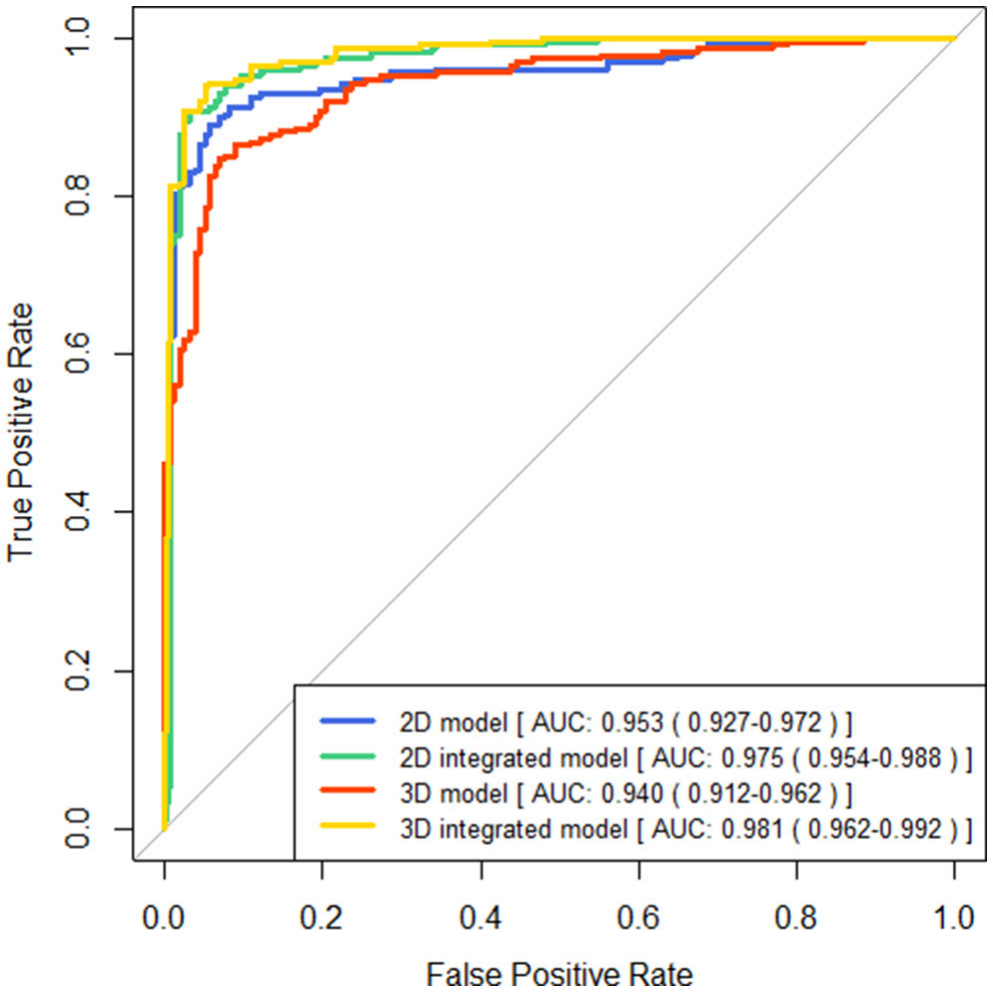
Comparisons of receiver operating characteristics (ROC) curves generated by brain atrophy models for two-type classification.

**Figure 5:**
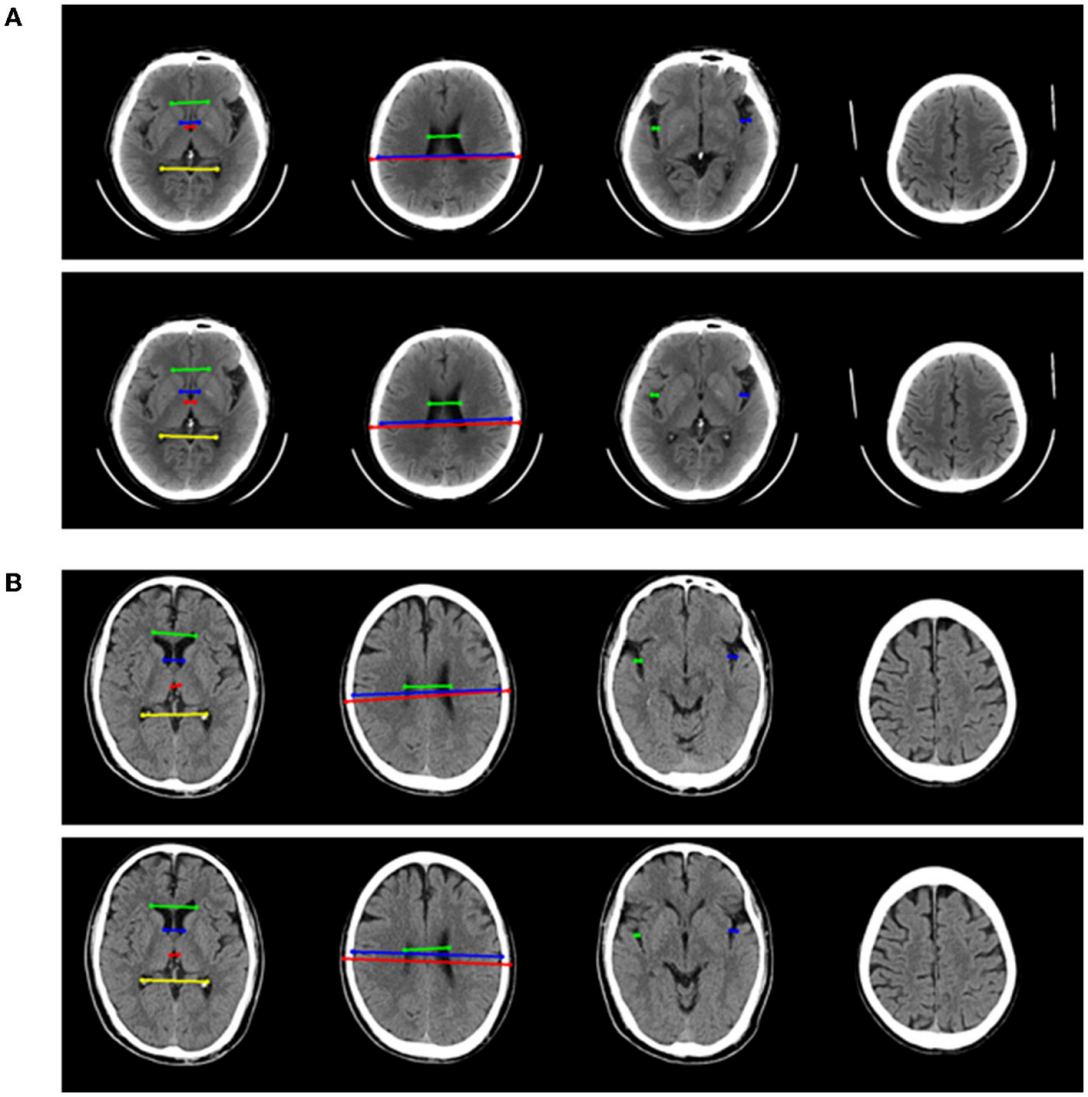
Two representative examples of atrophy cases, which were misclassified. For each example, four key slices were presented from manual annotation (top) and detection of 2D model (bottom). On key slice 1, the green line represented A, the blue line represented B, the red line represented C and the yellow line represented D; On key slice 2, the green line represented E, the blue line represented F and the red line represented G; On key slice 3 (*in Figures 5A,B), the green line represented HL (long arrows), while the blue line represented HR (long arrows). **(A)** Example case was misclassified by 2D model but was correctly predicted by 3D model, 2D-integrated model, and 3D-integrated model. **(B)**. Example case was misclassified by both 2D model and 3D model but was correctly predicted by 2D-integrated model and 3D-integrated model.

The performance of four models for two-type classification in the test set was assessed (Table 3 and Figure 4). The results showed that the 2D-integrated model yielded the highest sensitivity value (0.951), while the 3D-integrated model yielded the highest specificity value (0.943), accuracy value (0.943), F1 score (0.951), and AUC (0.981). Models with different strategies, all achieved great AUCs for two-type classification (>0.94). Furthermore, ROCs analysis showed the 2D model and 3D model, had no significant differences in statistics. Integration of age and gender information could significantly improve the model performance, while AUCs of the 2D-integrated model and 3D-integrated model had no significant differences (**Supplementary Table S2**). The representative cases of misclassifications showed key slice 3 (^*^in Figures 5A,B) detected by the 2D model was the adjacent slice to the manually annotated slice (reference) in both cases, which led to shorter H (long arrows) and might be the reason for misclassifications.

**Table 3:** Performance comparison for two-class classification of brain atrophy in test set, in terms of sensitivity, specificity, accuracy, F1 score, and area under the curve (AUC).

	**Sensitivity**	**Specificity**	**Accuracy**	**F1 Score**	**AUC**
2D model	0.931	0.854	0.899	0.916	0.953 (0.927–0.972)
2D integrated model	0.951	0.902	0.932	0.943	0.975 (0.954–0.988)
3D model	0.851	0.924	0.881	0.894	0.941 (0.912–0.962)
3D integrated model	0.943	0.943	0.943	0.951	0.981 (0.962–0.992)

*AUC presented as the value of AUC (95% confident interval)*.

### Performance of the Three-Type Classification for Brain Atrophy With CSVD

**Figure 6:**
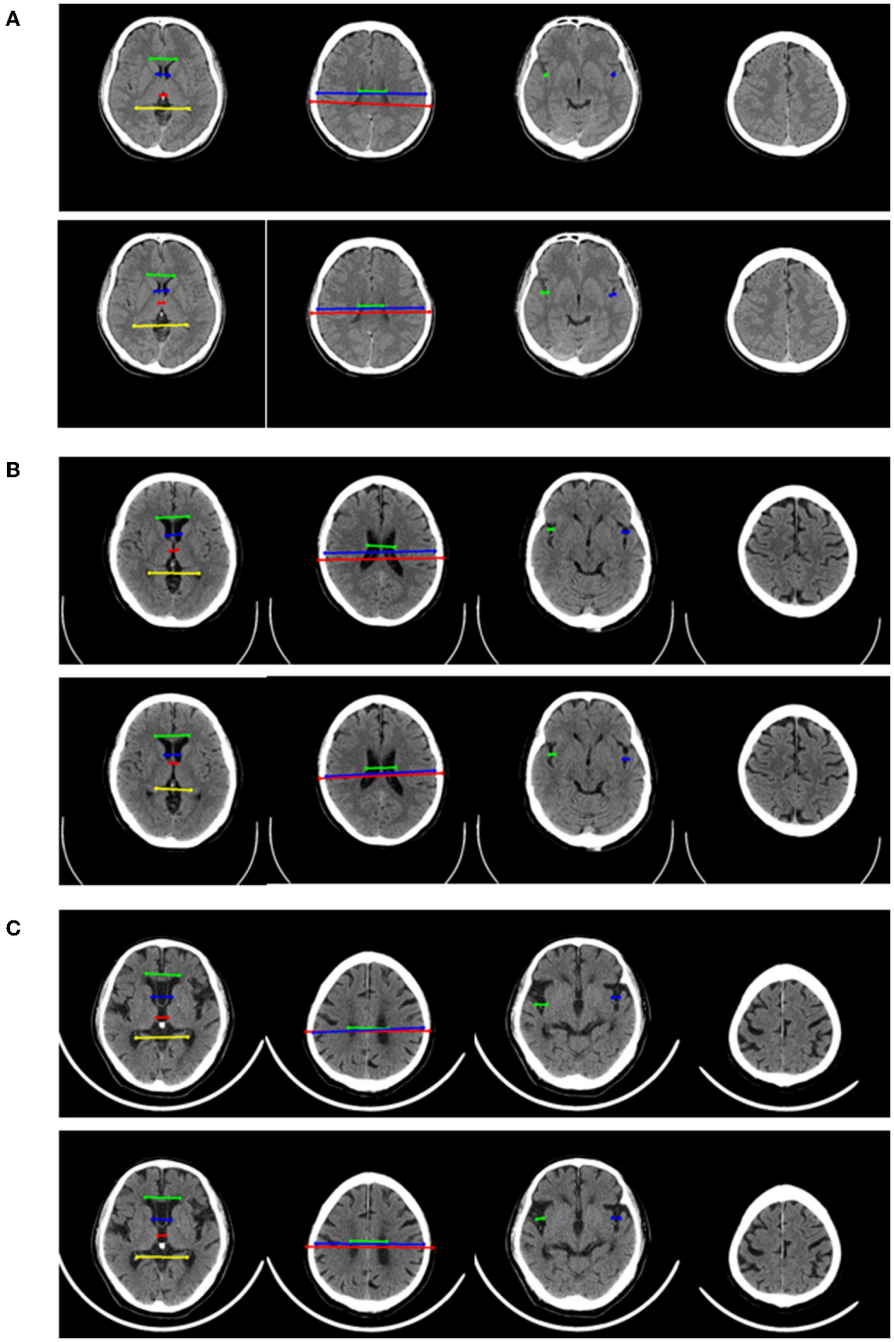
Representative examples in no-atrophy, mild atrophy, and severe atrophy groups that were misclassified. For each example, 4 key slices were presented from manual annotation (top) and results of 2D model (bottom). On key slice 1, the green line represented A, the blue line represented B, the red line represented C and the yellow line represented D; On key slice 2, the green line represented E, the blue line represented F and the red line represented G; On key slice 3, the green line represented HL, while the blue line represented HR. **(A)**. Example case that was misclassified by 2D model but correctly predicted by 3D model, 2D-integrated model and 3D-integrated model in the no-atrophy group. **(B)**. Example case that was misclassified by 2D model, 3D model and 3D-integrated model but correctly predicted by the 2D-integrated model in mild atrophy group. **(C)**. Example case that was misclassified by all four models in severe atrophy group.

The performance of four models for three-class classification tasks was shown (Table 4). In the test set, the 2D-integrated model achieved the highest accuracy (0.808) and kappa (0.786), while the 3D-integrated model showed the highest average F1 score. All models had a substantial agreement with experts in diagnosing and classifying the degree of brain atrophy. Furthermore, we evaluated the classification performance of no atrophy, mild atrophy, and severe atrophy, respectively (Supplementary Table S3). Consistent with the results of two-type classification, the models all achieved a high performance with the identification of no-atrophy subjects. The 2D-integrated model was the best-performed model with an accuracy of 0.932, F1 score of 0.917, and AUC of 0.978 (0.959–0.991). In mild and severe atrophy, the AUC of the 2D model was consistently higher than that of the 3D model. Integration of age and gender improved the AUC of the 2D model for mild atrophy classification but showed no significant difference from severe atrophy. Noticeably, all models achieved a low sensitivity (< 0.6) and a high specificity (>0.93) for severe atrophy. The representative cases showed a longer H and were achieved by the linear measurement of the 2D model in the no-atrophy patient (Figure 6A short arrows). In the mild atrophy case, a shorter D (short arrows in Figure 6A) was achieved and the cerebral sulci on key slice 4 was misidentified as normal while manually annotated as enlargement (Figure 6B^*^). In the severe atrophy case, shorter C and H (short arrows in Figure 6C) were achieved, which might be the reason for misclassification.

**Table 4:** Performance comparison for three-class classification of brain atrophy in test set, in terms of accuracy, average F1 score, and Weighted kappa coefficient.

	**Accuracy**	**Average F1 score**	**Kappa**
2D model	0.761	0.716	0.727
2D integrated model	0.808	0.74	0.786
3D model	0.73	0.657	0.607
3D integrated model	0.795	0.774	0.742

## Discussion

In this study, we developed and verified a new pipeline that is different from end-to-end. It is applicable to brain atrophy assessment, using linear measurement. On this basis, we provide a fully automatic brain atrophy classification model, which combines machine learning and deep learning, which can be applied to CSVD patients. In addition, by integrating limited clinical information and age and gender, the classification performance of the model is improved. It may be incorporated into clinical decision support tools in the future.

One of the motivations that drove our study is that brain atrophy has become an important risk factor for brain health with CSVD, and the effects of CSVD can be mediated through the development of brain atrophy (33). The increased white matter hypersignal burden undermines the integrity of the white matter and causes the loss of volume. CSVD-related cortical atrophy may result from secondary neurodegenerative processes elicited by degeneration to the WM tracts that disrupt the functional connections of the brain (8). In a longitudinal study, Jokinen H et al. visually assessed the severity of brain atrophy on MRI and confirmed that brain atrophy is independently related to cognitive decline in small vessel diseases (10). Medial temporal lobe atrophy (MTLA), subcortical and cortical atrophy increase the impact of white matter lesions (WML) and lacunae on cognitive decline. This evaluation conclusion was corroborated in another MRI study that used automated brain volume assessment (6). Brain atrophy is related to the adverse outcome of acute ischemic stroke after reperfusion therapy (34). It can be considered as an indicator of brain weakness and is more susceptible to ischemia (35). A study of automatic CT volume segmentation showed that brain atrophy reduces the possibility of functional recovery after acute ischemic stroke in the anterior circulation (36). Our cross-sectional study of a group of CSVD subjects supports that the automatic assessment of brain atrophy is realistically achievable. Accurate clinical information (presence/absence and severity) of brain atrophy can be obtained through rapid and fully automatic linear measurement in NCCT. In the test set, the classification performance of the 2D integration model reached a significant agreement with the expert consensus (kappa = 0.786). This may be a friendly and effective assistant for young clinicians, involving decision-making and prognosis of CSVD.

Another important and original intention of our research is to realize and verify the method of fully automatic assessment of brain atrophy on NCCT images, which is also the reason why other neuroimaging features of CSVD patients were not involved in the study. As far as we know, this pilot study is the first attempt to automate linear measurement for the assessment of brain atrophy on NCCT. One-dimensional linear measurement is an effective and economical method for evaluating brain structure (29, 37). The linear index indirectly reflects the structural information of different brain regions. Based on the measured “Hackerman Number” and “ventricular index” data can reflect the degree of central atrophy (37, 38). The width of the Sylvian cistern and the sulci is related to the thickness of the cerebral cortex (39, 40). Linearity measurement is also suitable for evaluating patients with CSVD. Qin calculated the Sylvian fissure ratio (SFR) on the MRI axial image (29). It was defined as the average of the maximal Sylvian widths taken from the cut showing the widest Sylvian fissure divided by the transperineal coronal inner table diameter. In elderly patients with CSVD, SFR has clinical value in screening cognitive decline. Our linear measurement covers the lateral fissure cistern of the brain as well. What's more, we not only automate this linear measurement, but also include more one-dimensional measurements reflecting the brain structure, which will help improve the accuracy of the evaluation and also facilitate its application in different clinical scenarios.

Although the evaluation of CSVD relies on more advanced neuroimaging, mainly MRI structural and functional imaging, CT still plays an important role in primary healthcare centers. Hanning U quantitatively assessed the leukoaraiosis on NCCT images successfully (17). Subsequently, Chen and Pitkänen used machine learning and convolutional networks to further quantify white matter lesions on NCCT (41, 42). Recently, Kaipainen completed the simultaneous assessment of white matter lesions and brain atrophy, but the automated training is very complicated and requires MRI as template mapping (43). Moreover, the ventricular system has not been integrated. As one of many attempts to automate the analysis of CT images, our study can be a useful supplement to the automated evaluation of CSVD. In view of the difference in imaging principles, CT has a lower soft tissue contrast than MRI, and accurate three-dimensional volume measurement is a huge challenge. The presence of WM lesions affected segmentation-based brain volume measurements on MRI (44). For patients with CSVD, as the burden of white matter lesions increases, the density of white matter around the ventricular system decreases, and the segmentation and calculation of ventricle-white matter tissue relying on the difference of the Hounsfield unit threshold brings greater difficulties (17). On the subjects with CSVD, linear measurement is assumed to avoid the interference of white matter lesions. It has been proven that there is a correlation between one-dimensional measurement and three-dimensional measurement results.

Two different models of the underlying system design are presented in this study, one is the end-to-end pipeline, which is considered advanced, and the other is a pipeline composed of several modules. Although the accuracy of key slices detectors failed to reach 100%, the automatically detected key slice is the adjacent slice of manual annotated one in the rest of the cases. The cross-sectional anatomy on the adjacent CT slices of each key slice is similar to the key slice of the ground truth Information of measurements and cerebral sulci can also be obtained on the adjacent slices. We fed three consecutive slices into three channels of the RGB color image to ensure the features of defied key slices can be acquired. Furthermore, three consecutive slices would provide more anatomical information and contribute to generate robust models for the measurement of ventricle indices. The final results show that it is feasible to establish an atrophy classification model based on different modules. For the binary classification, the 2D model shows relatively higher sensitivity and lower specificity than the 3D model. The AUC is 0.953 (0.927–0.972) and 0.941 (0.912–0.962), respectively. In the three-classification task, the Kappa values of the two models were 0.727 and 0.607, respectively. Although both 2D and 3D models have application value in the future, we believe that some of the advantages of linear measurement modules are unique. Different from end-to-end, it is considered a black box between input and output, and the linear side measurement pipeline is artificially divided into four modules. Each module has a specific function and is visualized. This is closer to the thinking logic of human medicine, and therefore, has a greater clinical interpretability. In addition, this strategy improves the predictability of the model output. Adding or reducing the corresponding modules flexibly according to the specific clinical instructions is also easier to achieve. This can also reflect the significance of this pilot study.

There are several limitations in this study. First, this is a retrospective single-center study with a small sample size. Insufficient data will lead to the overfitting problem of deep learning algorithms and limit model performance. Although we have adjusted the ratio of no-atrophy and brain atrophy, the dataset is still imbalanced for a three-type task. Hence, a large and standard dataset is needed to improve model performance. And model robustness should be verified on diverse external datasets in future studies. Second, the present study excluded the atrophy of the cerebellum and brain stem and mainly focused on the assessment of cerebral atrophy, because image artifacts often present in the posterior cranial fossa of NCCT which may interfere with linear measurements and mislead classification. Third, the collected clinical information is limited. More demographic information, medical test results and medical history may be integrated into present models. Lastly, the lack of performance comparison between the automated algorithms and physicians with different training levels is also a limitation. The performance of physicians should be investigated to further elucidate the usefulness of our models.

In conclusion, we develop an end-to-end 3D CNN model and an automatic pipeline by using deep learning and machine learning algorithms to predict brain atrophy degree. The 2D model yields equivalent high performance when compared with the 3D model in predicting the presence of brain atrophy, while the 2D model is superior to the 3D model in predicting the degree of brain atrophy. Integration of age and gender can significantly improve the performance for brain atrophy classification. Compared to the end-to-end 3D CNN model, the 2D model follows the linear measurements approach which is more comprehensible to clinicians. Therefore, the application of our 2D linear measurement-based pipeline has the potential to assist physicians to reduce variants and improve efficiency.

## Data Availability Statement

The raw data supporting the conclusions of this article will be made available by the authors, without undue reservation.

## Ethics Statement

The studies involving human participants were reviewed and approved by the Ethics Committee of the First Affiliated Hospital of Zhejiang University (2019.1511). Written informed consent for participation was not required for this study in accordance with the national legislation and the institutional requirements.

## Author Contributions

XZ, RJ, TZ, and TC collected clinical data of patients. JW, SC, HLi, HLo, and BX performed the image analysis. JW, SC, JY, and HLo analyzed the study data and build model. JW and SC wrote the paper. HLo, YZ, and JY reviewed and revised the manuscript. All authors contributed to the design of the study, gave advice, and reviewed the manuscript.

## Funding

This study has received funding from National Natural Science Foundation of China (No. 81871428).

## Conflict of Interest

JY was employed by the Taimei Medical Technology. The remaining authors declare that the research was conducted in the absence of any commercial or financial relationships that could be construed as a potential conflict of interest.

## Publisher's Note

All claims expressed in this article are solely those of the authors and do not necessarily represent those of their affiliated organizations, or those of the publisher, the editors and the reviewers. Any product that may be evaluated in this article, or claim that may be made by its manufacturer, is not guaranteed or endorsed by the publisher.
