# Supplementary material for: Fully Automatic Classification of Brain Atrophy on NCCT Images in Cerebral Small Vessel Disease: A Pilot Study Using Deep Learning Models
Source: Front Neurol. 2022 Mar 24;13:846348. doi: 10.3389/fneur.2022.846348 (PMC8989434; doi:10.3389/fneur.2022.846348)
Supplement: Supplementary file 1 [file Data_Sheet_1.docx]

**Supplementary Material**

Table S1 Key slice detection performance of key slice detectors in 2D model

|  | Acc0 | Acc1 |
| --- | --- | --- |
| Key slice 1 | 0.887 | 1 |
| Key slice 2 | 0.934 | 1 |
| Key slice 3 | 0.558 | 1 |
| Key slice 4 | 0.574 | 1 |

Acc0, the accuracy that the detected key slice is the manually annotated one; Acc1, the accuracy that the detected key slice is one of annotated key slice and its adjacent slices

Table S2 Statistical analysis of ROC curves of different models for two-class classification

|  | Difference | SD | 95% CI | Z statistic | *p* value |
| --- | --- | --- | --- | --- | --- |
| 2D vs 2D integrated | 0.022 | 0.006 | 0.011-0.034 | 3.71 | ＜0.001 |
| 2D vs 3D | 0.013 | 0.011 | -0.009-0.038 | 1.151 | 0.250 |
| 2D vs 3D integrated | 0.028 | 0.009 | 0.011-0.046 | 3.133 | 0.002 |
| 2D integrated vs 3D | 0.035 | 0.011 | 0.014-0.056 | 3.203 | 0.001 |
| 2D integrated vs 3D integrated | 0.006 | 0.005 | -0.004-0.015 | 1.184 | 0.236 |
| 3D vs 3D integrated | 0.041 | 0.011 | 0.019-0.062 | 3.717 | ＜0.001 |

SD, standard deviation

Table S3 Performance comparison of different models in no-atrophy, mild atrophy and severe atrophy group

|  | Model | Sen | Spe | Acc | F1 Score | AUC |
| --- | --- | --- | --- | --- | --- | --- |
| Normal | 2D | 0.866 | 0.930 | 0.904 | 0.880 | 0.957 (0.932-0.975) |
|  | 2D integrated | 0.911 | 0.947 | 0.932 | 0.917 | 0.978 (0.959-0.991) |
|  | 3D | 0.911 | 0.873 | 0.888 | 0.869 | 0.919 (0.887-0.944) |
|  | 3D integrated | 0.911 | 0.934 | 0.925 | 0.908 | 0.930 (0.900-0.953) |
| Mild atrophy | 2D | 0.861 | 0.723 | 0.782 | 0.772 | 0.877 (0.840-0.908) |
|  | 2D integrated | 0.879 | 0.759 | 0.810 | 0.799 | 0.917 (0.885-0.943) |
|  | 3D | 0.733 | 0.768 | 0.753 | 0.718 | 0.764 (0.719-0.806) |
|  | 3D integrated | 0.818 | 0.827 | 0.823 | 0.799 | 0.831 (0.790-0.867) |
| Severe atrophy | 2D | 0.365 | 0.978 | 0.878 | 0.494 | 0.936 (0.907-0.959) |
|  | 2D integrated | 0.381 | 0.975 | 0.878 | 0.505 | 0.937 (0.907-0.959) |
|  | 3D | 0.317 | 0.935 | 0.834 | 0.385 | 0.742 (0.696-0.785) |
|  | 3D integrated | 0.571 | 0.944 | 0.883 | 0.615 | 0.766 (0.721-0.808) |

Sen, sensitivity; Spe, Specificity; Acc, accuracy;

AUC presented as the value of AUC (95% confident interval).
